# Factors Motivating Traditional Healer versus Biomedical Facility Use for Treatment of Pediatric Febrile Illness: Results from a Qualitative Study in Southwestern Uganda

**DOI:** 10.4269/ajtmh.19-0897

**Published:** 2020-05-26

**Authors:** Anneka Hooft, Doreen Nabukalu, Juliet Mwanga-Amumpaire, Michael A. Gardiner, Radhika Sundararajan

**Affiliations:** 1 Department of Pediatrics, Rady Children’s Hospital San Diego, University of California, San Diego, San Diego, California;; 2 Department of Community Health, Mbarara University of Science and Technology, Mbarara, Uganda;; 3 Department of Paediatrics and Child Health, Mbarara University of Science and Technology, Mbarara, Uganda;; 4 Department of Emergency Medicine, Weill Cornell Medicine, New York, New York;; 5 Center for Global Health, Weill Cornell Medicine, New York, New York

## Abstract

Febrile illnesses, such as malaria and pneumonia, are among the most common causes of mortality in children younger than 5 years in Uganda outside of the neonatal period. Their impact could be mitigated through earlier diagnosis and treatment at biomedical facilities; however, it is estimated that a large percentage of Ugandans (70–80%) seek traditional healers for their first line of medical care. This study sought to characterize individual and structural influences on health care–seeking behaviors for febrile children. Minimally structured, qualitative interviews were conducted for 34 caregivers of children presenting to biomedical and traditional healer sites, respectively. We identified six themes that shape the pathway of care for febrile children: 1) peer recommendations, 2) trust in biomedicine, 3) trust in traditional medicine, 4) mistrust in providers and therapies, 5) economic resources and access to health care, and 6) perceptions of child health. Biomedical providers are preferred by those who value laboratory testing and formal medical training, whereas traditional healer preference is heavily influenced by convenience, peer recommendations, and firm beliefs in traditional causes of illness. However, most caregivers concurrently use both biomedical and traditional therapies for their child during the same illness cycle. The biomedical system is often considered as a backup when traditional healing “fails.” Initiatives seeking to encourage earlier presentation to biomedical facilities must consider the individual and structural forces that motivate seeking traditional healers. Educational programs and cooperation with traditional healers may increase biomedical referrals and decrease time to appropriate care and treatment for vulnerable/susceptible children.

## INTRODUCTION

Febrile illnesses remain the most common cause of mortality in children younger than 5 years in Uganda outside of the neonatal period.^1–3^ These include potentially treatable infections such as pneumonia, malaria, and diarrheal disease.^3^ Delays in diagnosis and treatment of these treatable illnesses directly contribute to pediatric morbidity and mortality.^1,4–6^ Most of pediatric deaths in Uganda do not occur in biomedical facilities,^7^ illustrating that the sickest children are often managed outside of the formal healthcare system. To reduce pediatric morbidity and mortality from curable diseases, there must be a better understanding of how caregivers manage illness among high-risk children outside of biomedical facilities.

Care-seeking behavior of caregivers of febrile children in Uganda and other areas of sub-Saharan Africa is variable and not well understood.^8–11^ Despite government-funded biomedical health facilities being free, larger referral hospitals where resources are concentrated tend to be less accessible for those living in rural areas. Local government-funded clinics have limited testing capabilities and supplies, whereas private clinics vary in quality and are largely cost-prohibitive to all but Ugandans of the highest socioeconomic status.^12^ Uganda is a medically pluralistic context, and caregivers of sick children may use informal healthcare resources, such as traditional healers^13–16^ and unlicensed drug shops.^6,17–20^ These informal resources are sought concurrently with, or instead of, biomedical resources.

A Ugandan Ministry of Health report estimated that the over 60% of caregivers with children younger than 5 years did not seek biomedical care for malaria treatment within 24 hours of symptom onset.^21^ This delay to initiation of biomedical treatment is influenced by multiple factors, including caregivers’ understanding of the cause of illness, perceived need of when the child is sick enough to warrant evaluation, and structural variables such as lack of transportation, economic and food insecurity, accessibility of health facilities, and familial or professional obligations.^21–28^ Pluralistic health behavior can further delay biomedical diagnosis and treatment,^6,22–26,29–32^ as severe symptoms of malaria and other illnesses are sometimes still attributed to traditional or spiritual causes and thought to be outside of the scope of care of biomedical health facilities.^4,26^

It is not well understood if there are fundamental differences in the perception of febrile illness between caregivers who choose to seek traditional healers and those who prefer to use biomedical facilities, and which factors most strongly impact health-seeking behavior. This gap in knowledge prevents culturally appropriate, effective interventions to improve clinical outcomes for children with febrile illness in medically pluralistic settings. The goal of this qualitative study was to identify factors that influence engagement with both formal and informal healthcare resources among caregivers of children with febrile illness in rural southwestern Uganda.

## MATERIALS AND METHODS

### Study location.

The district of Mbarara is located approximately 250 km southwest of the Ugandan capital city of Kampala, with a population estimate of 470,000. Mbarara is a malaria-endemic area with peaks of severe malaria during rainy months, which are primarily between the months of September through January and March through May.^33^

### Study sites.

Participants were enrolled at one of six sites within the town of Mbarara. We recruited participants at three biomedical sites: Mbarara Regional Referral Hospital (MRRH) Toto Inpatient Ward, MRRH Outpatient Department, and Mbarara Municipality Clinic IV (MMC). Both MRRH and MMC are located within Mbarara town within one kilometer distance. Mbarara Regional Referral Hospital is a 600-bed facility serving more than 25,000 outpatients monthly and admitting about 100 patients daily, 20% of whom are children. The pediatrics department has approximately 70 inpatient beds with access to resuscitation equipment, oxygen, and various diagnostic laboratory tests and admits 5,000–8,000 children annually. Mbarara Municipality Clinic is an outpatient clinic for children and sees between 200 and 250 children per month, with an average volume of 10 patients per day. The clinic runs from Monday to Saturday each week. All three biomedical recruitment sites are public, government-funded health facilities.

We also recruited participants at three traditional healer sites: a commercial entity approximately 2 km from the town center which sees up to 50 pediatric patients per week, a clinic approximately 5 km from the town center which sees approximately eight pediatric patients per week, and another clinic located approximately eight kilometers from the town center which sees approximately 20 pediatric patients per week. The traditional healer sites used for study recruitment are all practicing herbalists, who primarily evaluate and treat patients with traditional herbal remedies.

This study was a sub-study of a multiyear National Institutes of Health-funded study on traditional healer utilization in Mbarara district (K23MH11409, 2017–2021). Based on experience conducting research in the region, study staff determined herbalists were most commonly sought for consultation by caregivers of children suffering from acute illness (as opposed to other types of traditional healers which include spiritual healers, bonesetters, and traditional birth attendants). We identified three healers with a reputation for seeing or specializing in pediatric illness to maximize recruitment of caregivers presenting with children at these sites. Additional criteria used to define traditional healer sites for recruitment were according to UNAIDS guidelines: 1) persons recognized by the local community as healers, 2) having regular patient attendance, and 3) having space to receive and treat patients.^34^

### Sampling, recruitment, and enrollment.

We used purposive sampling to identify participants, identifying key informants with knowledge and experience pertaining to healthcare-seeking behavior for children. Potential participants were caregivers of children seeking health care for a febrile child younger than 12 years. Participants were recruited on conclusion of their visit for a child presenting for evaluation of febrile illness or during their admission to the pediatric ward, so as not to impede the normal flow of patient care. Criteria for caregiver inclusion included age of 18 years or older, a caregiver of a child younger than 12 years, and ability to provide consent to participate in the study in one of the two languages spoken by study staff (English or the local language, Runyankole)*.* Exclusion criteria included incapacity to give informed consent and unwillingness to participate in the interview. Participants were provided a small household staple valued at 5,000 UGX (∼$1.50 USD) for their participation on conclusion of the interview.

In cases where literacy was limited, consent forms were read aloud. Enrollment was completed in a private area, either in a separate room located within the traditional healer or biomedical clinic, or in an office near the medical ward. Written consent was obtained from all participants.

Participant enrollment was continued until saturation was reached. We considered data saturation as the point when transcripts no longer revealed any substantial new or relevant information, and thematic development was sufficient based on the content contained in completed interviews,^30^ at which point study enrollment was considered complete (see “Data Analysis” for information on author engagement with transcript dataset).

### Data collection.

Interviews of approximately 1 hour in length were conducted between October 2018 and March 2019 by the coauthor D. N. Interviews were conducted in either Runyankole or English, depending on the participant’s preference. A semi-structured interview guide was used for consistency and to maintain focus on key topics, while still allowing for exploration of new or novel concepts. Interviews were audio recorded and then subsequently translated and transcribed into English by the interviewer.

### Ensuring data quality.

Integrity of transcription and translation was verified by one of the coauthors fluent in both languages used in the study (J. M. A.). Transcripts were also monitored by A. H. and R. S. for clarity during the data collection period, with attention to detail, grammar, and style.

### Data analysis.

Data were analyzed by A. H. and R. S. using an inductive approach and thematic analysis framework.^35^ All transcripts were reviewed by both authors within 72 hours of completion to assess for content, identify topics of convergence and divergence with other interviews, and conduct the process of data reduction. After approximately half of the interviews had been completed, A. H. and R. S. generated an initial list of codes to share with the research team (D. N., J. M. A., and M. G.). Following team discussion, codes were modified and regrouped. Additional interviews were conducted to explore incomplete or emerging concepts. Atlas software (ATLAS.ti Scientific Software Development, GmbH, Berlin, Germany) was used to organize study data and codes but not in the generation of codes.

After an initial set of codes was produced, we observed that emerging content paralleled factors which have been well described in Andersen’s model of healthcare utilization.^36^ Andersen’s model was originally created to aid in understanding utilization of health services and in the development of systems aimed at improving equitable access to care. The model accounts for individual factors, community-based factors, and structural factors that influence when and where health services are sought.^36,37^

Using the framework provided of Andersen’s model of healthcare utilization, we organized content into categories corresponding to predisposing community- and personal/family-based factors that guide health care–seeking behavior.^36,37^ These categories were refined to generate an explanatory model for pathways and influences on health care–seeking behaviors specific to our study population and the medically pluralistic context. Through careful, repeated examination and iterative review of the dataset, interpretation, and grouping of codes, we identified six themes that are central to the health care–seeking pathway for febrile children in our study population.^38^ Participant quotations illustrating these themes were then selected from the dataset and are presented here to demonstrate concepts relevant to our study.

### Ethical considerations.

This study was approved by the Weill Cornell Medicine Institutional Review Board (Protocol 1803019105), the University of California, San Diego Institutional Review Board (Protocol 170672), the Mbarara University of Science and Technology Institutional Review Board (Protocol 16/01-17), and the Ugandan National Council on Science and Technology (Protocol SS4338). All interviews were conducted in secure, private locations to maintain participant confidentiality.

## RESULTS

### Sample characteristics.

A summary of characteristics of caregiver participants is shown in Table 1; characteristics were largely similar between caregivers at healer and biomedical sites. Of 46 eligible caregivers approached, 12 declined to participate. Most refused because of time limitations, with a few refusing because of inability to care for or soothe their child during the interview. Of the 12 refusals, seven occurred at biomedical sites and five occurred at traditional healer sites. A total of 34 caregivers were included in this study, the majority of whom were female (*n* = 32, 94%). An equal number of participants were interviewed in each group: 17 healer clients (one male and 16 female) and 17 biomedical clients (one male and 16 female). Caregivers’ age ranged from 19 to 57 years. The age of the child being evaluated ranged from 2 weeks to 11 years, with a mean age of approximately 2 years for both groups. Most caregivers had completed primary or secondary school, with a few having completed postsecondary diplomas. Most caregivers were the mothers (*n* = 28) of the children being evaluated, but there were also fathers (*n* = 2), grandmothers (*n* = 2), one neighbor, and one cousin (Table 1). One interview was conducted in English (a healer client who was the father of a child), at the participant’s request, with the remainder conducted in Runyankole.

**Table 1:** Summary of participant characteristics

Characteristic	Healer patients (*n* = 17)	Biomedical patients (*n* = 17)
Female caregiver, *n* (%)	16 (94)	16 (94)
Caregiver age (years), mean (SD)	30.8 (8.5)	29.1 (9.1)
Caregiver relationship with the child, *n* (%)	Mother, 13 (76.5)	Mother, 15 (88.2)
	Grandmother, 1 (5.9)	Grandmother, 1 (5.9)
	Father, 1 (5.9)	Father, 1 (5.9)
	Cousin, 1 (5.9)	
	Neighbor, 1 (5.9)	
Highest level of education, *n* (%)	None, 1 (6)	None, 1 (6)
	Primary school, 6 (35)	Primary school, 6 (35)
	Secondary school, 6 (35)	Secondary school, 5 (29)
	Diploma or higher, 4 (24)	Diploma or higher, 5 (29)
Child’s age (years), mean (SD)	2.0 (2.6)	1.6 (1.7)
Male child, *n* (%)	7 (50)	10 (71)

### Qualitative results.

**Figure 1.**
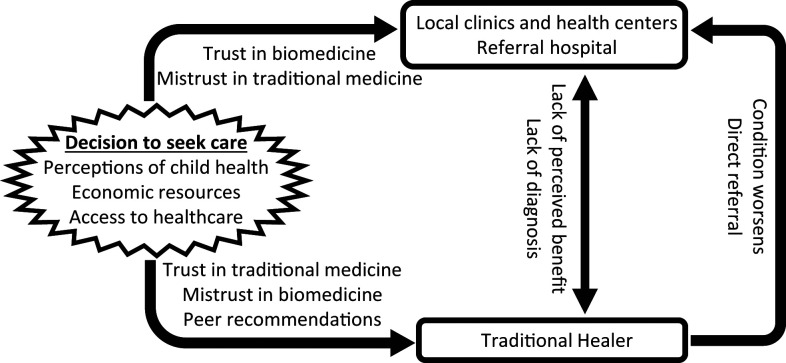
Factors influencing health care–seeking behavior at traditional healer and biomedical sites.

We identified six main themes that shape the pathway of care for febrile children: 1) peer recommendations, 2) trust in biomedicine, 3) trust in traditional medicine, 4) mistrust in providers and therapies, 5) economic resources and access to health care, and 6) perceptions of child health (Figure 1).

### Peer recommendations.

Peer recommendations are strongly considered in the decision as to where to seek health care for a febrile child. Caregivers are given advice from spouses, parents, extended family, local elders, friends, and neighbors. While both biomedical and healer participants describe the process of soliciting guidance in determining when and where to seek care, peer recommendations more frequently influence where caregivers bring children to receive traditional treatments. A mother at a biomedical clinic describes how advice from friends and neighbors helps direct which traditional healer she visits for care when her child is ill.

“Some [healers] are trustworthy, but not all. There are those ones who have experience and are known. For example, if your child has a problem, your friends or neighbors can direct you to go to a certain healer. If about four people direct you to one healer, then you can tell that that person is trustworthy.” (Biomedical client, mother, age 24 years)

### Trust in biomedicine.

Caregivers who primarily use biomedical facilities seek advice from others when their child is ill but more often prefer to go directly to the hospital and obtain evaluation from a trained medical professional. This mother describes why she prefers her child to receive care from biomedical health workers.

“When the child gets sick, most of the time, I don’t go around asking people for advice. I don’t ask for anyone’s opinion, that maybe the child has the following signs. What’s your advice? No, I don’t do that. Because even the people at home don’t know anything about treating children. So, for me I go to the hospital and ask a health worker, because I’m sure they know all about the disease conditions and have studied them. Now, if you consulted me that your child has a fever and headache and you want me to tell you what the problem is, I can’t help you because I don’t know. I didn’t study that. What advice do you expect me to give you? … so, I don’t ask anyone. I just go to the hospital.” (Biomedical client, mother, age 21 years)

Some caregivers prefer biomedical providers rather than traditional healers primarily because of their ability to provide laboratory or radiologic testing to guide diagnosis and treatment. One mother describes the peace of mind that comes from having testing performed at a biomedical facility:

“For me, I just go to the hospital. Here I know they will at least do some blood checkup. Even if it’s just for a few diseases that they check, that’s better because they would have screened out some.” (Biomedical client, mother, age 21 years)

Many caregivers reported alternating use of both healthcare systems. For example, caregivers described getting specific conditions such as malaria ruled out first at the biomedical facility through laboratory testing and then going to consult a healer for further care. This mother describes her initial preference for biomedical testing:

“I prefer going to the hospital first. If they tell me that they cannot find the problem after carrying out tests on his blood and doing other body checkups, then I can try going to the traditional healers.” (Healer client, mother, age 37 years)

### Trust in traditional medicine.

Caregivers seeking care from both biomedical and traditional providers described specific traditional diseases such as “neonatal teeth,” seizures, and “millet chest,” of which diagnosis and treatment is considered outside the scope of biomedical providers. This mother describes symptoms in her child, which she believes are specific to the local culture (Kinyankole).

“Sometimes children suffer from conditions which do not require treatment from the hospital, but healers. My child is suffering from ebihungu (seizures) which is a Kinyankole disease and does not need to be taken to hospital. That’s where a traditional healer comes in to help. Some diseases are traditional and cannot be treated with modern medicine. The biomedical workers do not know ebihungu.” (Healer client, mother, age 27 years)

Healer clients perceived little harm in trialing herbal therapy for febrile children. In some cases, the biomedical system was considered a backup for many caregivers of febrile children who prefer to receive initial care from a traditional healer:

“If you have stomach pain, there is good traditional medicine for stomach pain… it depends on the condition of the child. You give traditional medicine and the child does not improve, then you take the child to hospital … I start by using traditional medicine and then if the child does not improve, I come to the hospital.” (Biomedical client, father, age 23 years)

Many caregivers had familiarity with traditional treatments as a child that had been provided care at home by elders in the family. Healers could serve as a surrogate for an experienced family member, and there was comfort in using herbal therapies in comparison to other treatments:

“It was herbal medicine. I got it from the village. It’s my grandmother who gave it to me but when it got over, the problem came back... it had gone away with traditional medicine from my grandmother. But now that the problem is back and I’m not at my village, I have decided to come to [this healer]. I’m sure she will do something to help me.” (Healer client, mother, age 27 years)

Other caregivers had developed friendships with traditional healers and consider them respected members of the community. They trust the healer to provide sound advice on where the child would receive the best care, even if that means recommending that the child seek care elsewhere or referring a very sick child directly to the hospital. These caretakers receiving care from traditional healers describe their trust in the healer to refer them to biomedical care if traditional remedies fail.

“I decided to bring her to this healer. She is a very good lady and if she can’t manage the disease, she can let you know, and you look for other options… [my child] really improved. Ever since this healer started treating [child], she became my friend. (Healer client, mother, age 30)

“Every medicine that [this healer] gives works very well. Even if you come with syphilis or stomach pain, her medicine works very well. And when she fails, she can refer you to the main hospital. She even gives a referral letter to go with. Maybe If she sees that you don’t have any serious complication, she can work on you.” (Healer client, neighbor [female], age 39 years)

### Mistrust in therapies and providers.

Caregivers describe mistrust of both biomedical and traditional treatments. Biomedical facilities were often avoided because of concerns of corruption. Caregivers reported that clinics would give false results to sell expensive treatments and/or sell medications for a substantial markup:

“Some [clinics] may use fake chemicals, or if they have a clinic and a laboratory, they can tell you that you have malaria so that you start paying for the malaria treatment. So, because they want money, they end up doing things which are not genuine... they test people and tell them they have typhoid, brucella, malaria, and keep selling medicine to the person even when they are treating a wrong disease.” (Healer client, father, age 36 years)

“Those small clinics don’t even have a laboratory. They just depend on what you explain to them and then prescribe for you the medicines. Sometimes they will be lying. If he wants to cheat you, he will say that you have malaria and then you give him the money for the medicine.” (Biomedical client, mother, age 31 years)

Caregivers presenting to biomedical facilities also describe mistrust of traditional healers because of the belief that healers are motivated by money, rather than by helping their patients. Participants describe reports of healers giving the same treatment for every condition, prescribing treatments they knew were not appropriate, extending treatment duration, and/or trying to sell new regimens after initial therapy failed to make money. A mother describes her mistrust of traditional healers, which is rooted in these suspicions:

“So, for me, healers are not good. It’s better to go to the hospital and they treat the real disease that they have checked, and the person heals. Not those things of getting some herbs for relief, then after one week you fall sick again, then you go back, meanwhile you’re spending money and wasting your time. Of course, they keep encouraging you to keep drinking more jerrycans [bottles] of the herbs. The healer might tell you, last time you took a small bottle, now you take a big one. Because the more liters of herbs you take, the more money they make.” (Biomedical client, mother, age 21 years)

Despite the common view that herbal medications are safe and familiar, there were a few biomedical clients who mistrust herbal remedies because of concerns over safety and their unregulated nature.

“You see, the healer just gets their herbs and without cleaning them, boils and just gives you to drink. So, you just take without knowing what they treat. You even can’t be sure of the dosage, but you just give the herbs to the baby, which may put the baby in more danger.” (Biomedical client, mother, age 34 years)

### Economic resources and access to health care.

Traditional healer locations are frequently preferred because they are closer in proximity and easier to reach for those living further from the larger clinics or hospitals:

“I have just told you about my child. She was sick and in the middle of the night, it was a traditional healer who helped me. I did not go to a hospital. I won’t lie to you. I can’t leave my garden and go to hospital when this healer is here. No, no, please. For me I find it easy coming to a traditional healer compared to going to a hospital. It’s very near. And I can come here any time.” (Healer client, grandmother, age 56 years)

Although care at the government-funded facilities are free, caregivers must still be able to travel to facilities, losing time that could be spent generating income or caring for other family members. Both biomedical and healer clients acknowledged that healers allow more flexibility by accepting alternative payments, exchanging services for food items or goods, and offering repayment plans in small increments over time:

“Sometimes it’s because of the money. Some people don’t want to spend money going to hospitals when there is a healer nearby. Yes, [the healers] ask for money, but in most cases, they have a payment agreement. The person can be allowed to pay in installments depending on how the person earns. So instead of someone spending a week in the hospital, while buying food, they rather be in their homes and keeping fetching herbs from a healer. So, they keep working and don’t have to spend a lot.” (Biomedical client, mother, age 34 years)

Biomedical facilities are sometimes avoided because they are considered to have “hidden costs.” These include needing to buy medications or medical supplies and time spent waiting for care. This mother described having to buy supplies while her child was admitted as an inpatient:

“[In the hospital] I think I had to spend so much on syringes [to administer medication to my child].… at the pharmacy it’s five hundred shillings, but they also sell them here on the ward. The health workers sell them. You give them money and they get them for you. You give them the money in hiding so that you’re not caught. They also don’t want to give you the syringes when people are seeing in the open. When you pay her the money, she tells you to go to your bed and she brings them for you.” (Biomedical client, mother, age 19 years)

### Perceptions of child health.

Caregivers of children do not use thermometers to objectively measure fever. They rely on instinct and experience to guide them in distinguishing when their child is ill and in need of attention by a healthcare provider. While motivated by their observation of the child’s clinical condition, caregivers may not have specific knowledge of the illness etiology or which treatment modality would be most effective:

“You know when your child is sick, you keep on trying very many options because you badly want to see them well. That’s why we end up going everywhere, including going to the priests at church.” (Biomedical client, mother, age 24 years)

Caregivers receiving treatments from both biomedical and traditional providers felt that they had not received clear explanation of what treatment their child was given and for which condition. Frequently, they describe a lack of understanding of other possible causes of the child’s illness, beyond malaria infection:

“I’m not a health worker. I cannot know what causes it. I also just see a child getting fever. It could be because of an infection. They sometimes tell us in hospitals that it’s either malaria or an infection. But I also don’t know what they be meaning by infection.” (Healer client, mother, age 27 years)

“We are told that what causes fever among children are mosquitoes, however, we sleep under a mosquito net. Even the baby sleeps under a mosquito net… …No, [I don’t have any idea about other infections], for me I know that all fever is due to malaria.” (Biomedical client, mother, age 29 years)

## DISCUSSION

Our study identified several factors that influence care-seeking behaviors for febrile illness in children at both biomedical and traditional health facilities. Belief in traditional illnesses was commonly reported, even by those who primarily prefer to use biomedical facilities. Most caregivers concurrently seek multiple healthcare providers and treatment modalities until there is a perceived benefit. Traditional healers are often viewed as more convenient, accessible, and cost-efficient, whereas the biomedical system is believed to be a safety net if the child’s illness worsens, with referral hospitals considered more trustworthy than smaller, local clinics.

These findings are consistent with the existing literature demonstrating medical pluralism in Uganda is common and belief in traditional illness is pervasive.^13–16,18–20,39^ Despite knowing that caregivers may use both formal and informal medical systems, our results demonstrate that this behavior often occurs within the same illness cycle until there is a perceived benefit. A traditional healer or biomedical provider may be the first health worker to see the child, or they may be seeing the child much later in their illness trajectory, after care has already been sought elsewhere. This may lead to attribution of illness resolution in self-limited disease to the last provider seen or last treatment administered and ultimately influence future care-seeking preferences. As in prior studies, attributing symptoms of severe malaria or other biomedical illnesses to traditional causes is common and can lead to delays in definitive management.^4,28,40^

Healer accessibility and flexibility with payment are important factors driving preference of traditional treatments, despite government-funded clinic services being “free of charge.” Healers are consulted for more than just provision of herbal therapies; they are considered friends, elders, and respected members of the community.^15,20,41,42^ Healers with a reputation for caring for children are sought after and will even insist on malaria testing before treating a febrile child or refer sick children directly to the hospital. These behaviors have been described in other studies.^43^ Our data demonstrate that traditional healers are not ignorant to biomedical concepts of disease and consider biomedical etiologies as they provide health care for febrile children. Furthermore, participants report that healers are respected for recognizing the limits of their practice and referring for biomedical testing. These findings suggest that cooperation and communication between traditional and biomedical systems may be feasible and beneficial to patients.^44^ Further studies are needed to determine if both biomedical providers and traditional healers are willing to integrate to provide collaborative care.

Study participants expressed frustration with a lack of explanation given by healthcare providers as to the cause of the child’s illness and necessary treatment. In prior studies, biomedical providers have discussed how caregivers often ask for a specific diagnosis once malaria testing is negative or continue to demand treatment even when it is not indicated.^45^ Our work indicates that it may be beneficial to expand culturally sensitive education to communities on self-limited childhood illness and stress the importance of early malaria testing in children with fever. As health knowledge alone does not directly lead to changes in care-seeking behavior, there may also be a role for traditional healers to deliver appropriate health advice, supportive care instructions, and health information directly to caregivers.

Our study has a few limitations. As participants were recruited at traditional healer clinics and biomedical facilities relatively near Mbarara town center, we may have overlooked those children with the most difficulty in accessing medical care. Thus, our data may exclude additional challenges these caregivers face when seeking care for a critically ill child. Most participants were female, so additional perceptions on health-seeking behaviors by males in the family that may influence care may not have been fully elucidated. However, the high prevalence of females indicates that they are the caregivers who usually bring the child for treatment and evaluation. We did not note any significant thematic distinctions from the few male participants in this study. Finally, our biomedical sample was recruited from a public, government-funded clinic, and therefore, the experiences of caretakers receiving care at private biomedical clinics may not be reflected in our sample.

## CONCLUSION

Acute infections remain a significant cause of morbidity and mortality in susceptible children. We identified social and structural factors that influence caregiver preference for care-seeking at traditional healers and biomedical facilities for children with febrile illness. Like much of sub-Saharan Africa, the concurrent use of both health systems is common. Cooperation and communication between traditional healers and biomedical providers may be a practical way to reduce delays in definitive management for critically ill children.
